# Impact of Computed Tomography-Defined Osteopenia on Outcomes of Transcatheter Aortic Valve Implantation: A Single-Center Retrospective Study

**DOI:** 10.3390/jcm14207182

**Published:** 2025-10-11

**Authors:** Hiroshi Kurazumi, Ryo Suzuki, Takato Nakashima, Ryosuke Nawata, Toshiki Yokoyama, Kazumasa Matsunaga, Yosuke Miyazaki, Atsuo Yamashita, Takayuki Okamura, Akihito Mikamo, Motoaki Sano, Kimikazu Hamano

**Affiliations:** 1 Division of Cardiac Surgery, Department of Surgery and Clinical Science, Yamaguchi University Graduate School of Medicine, Ube 755-8505, Yamaguchi, Japan; kurazumi@yamaguchi-u.ac.jp (H.K.);; 2 Division of Cardiology, Department of Medicine and Clinical Science, Yamaguchi University Graduate School of Medicine, Ube 755-8505, Yamaguchi, Japan; 3 Department of Anesthesiology-Resuscitology, Yamaguchi University School of Medicine, Ube 755-8505, Yamaguchi, Japan

**Keywords:** aortic stenosis, transcatheter aortic valve implantation, osteopenia, frail, Japan

## Abstract

**Background/Objectives**: Transcatheter aortic valve implantation (TAVI) is a standard treatment for severe aortic stenosis, especially in older adults and high-risk patients. However, many TAVI candidates have osteopenia or osteoporosis, indicated by low bone mineral density (BMD), which is linked to frailty and adverse outcomes. Although prior research suggests an association with poor clinical outcomes, data remain limited. We investigated the impact of osteopenia on TAVI outcomes, hypothesizing that a lower BMD is associated with poor perioperative outcomes and decreased long-term survival. **Methods**: In this single-center retrospective study, we analyzed data from 411 patients who underwent TAVI at Yamaguchi University Hospital from 2014 to 2024. Clinical and survival data were collected, and Cox regression analysis was used to identify independent predictors of mortality. Preoperative BMD was measured using computed tomography, defining osteopenia as <135 Hounsfield units at L1. Patients were categorized as having mild, moderate, or severe osteopenia. **Results**: Early clinical outcomes and procedural success were similar; however, patients with osteopenia had longer intensive care unit stays (*p* = 0.04) and higher late cardiac mortality (*p* < 0.001). Six-year survival was 36.2% and 88.1% in patients with and without osteopenia, respectively (*p* < 0.0001). Cox regression analysis revealed osteopenia as a mortality risk factor (hazard ratio: 6.75, 95% confidence interval: 2.96–15.38, *p* < 0.0001). Severe osteopenia was associated with the poorest outcomes. **Conclusions**: Osteopenia is an independent predictor of poor long-term survival after TAVI. These findings underscore the importance of comprehensive risk assessment, suggesting that targeted interventions may improve outcomes.

## 1. Introduction

Severe aortic stenosis is increasingly prevalent in the context of global population aging. Surgical aortic valve replacement (SAVR) has long been considered the definitive treatment; however, in older patients and patients with multiple comorbidities, the procedure can carry considerable risk. Transcatheter aortic valve implantation (TAVI) has emerged as a less invasive alternative, and its efficacy and safety have been demonstrated, particularly in high-risk patient groups [1]. Recently, its indications have been expanded to include intermediate-risk and low-risk cases [2].

Although the indications for TAVI are gradually being expanded to include low-risk cases, most patients who undergo TAVI are still older and at high risk. A substantial subset of these patients of advanced age exhibit reduced bone mineral density (BMD)—a hallmark of osteoporosis or osteopenia. Low BMD not only increases the risk of fractures but has also been linked to sarcopenia and frailty [3], which may significantly influence long-term clinical outcomes in patients with cardiovascular disease [4,5].

Although the concepts of frailty assessment and comprehensive risk stratification have gained momentum in TAVI research, the specific impact of low BMD on perioperative and long-term outcomes remains understudied. Therefore, this study aimed to clarify the influence of low BMD on clinical outcomes in patients undergoing TAVI. We hypothesized that lower BMD might be associated with worse perioperative outcomes and decreased long-term survival. Our findings could inform patient selection strategies, perioperative management protocols, and the development of targeted frailty interventions in the TAVI population.

## 2. Materials and Methods

### 2.1. Study Population

This retrospective study included 411 consecutive patients who underwent TAVI for symptomatic severe aortic stenosis at Yamaguchi University Hospital (Ube, Japan) from 2014 to 2024. All patients who underwent TAVI for aortic valve stenosis, regardless of age, were enrolled in this study. The indications for TAVI were discussed at a multidisciplinary heart team conference and were primarily determined in accordance with Japanese guidelines [6]. This retrospective study was approved on May 9, 2024, by the Institutional Review Board of Yamaguchi University Hospital (Study ID: H2024-014). The research protocol complied with the latest version of the Declaration of Helsinki (including the World Medical Association Declaration of Taipei) and the “Ethical Guidelines for Life Science and Medical Research Involving Human Subjects” (revised in 2022). The requirement for written informed consent was waived based on the Ethical Guidelines for Medical and Biological Research Involving Human Subjects. Information on the study is publicly available on the website of Yamaguchi University School of Medicine Hospital; patients or their legal representatives who might have participated in the study had the opportunity to refuse participation.

### 2.2. CT-Based Assessments of BMD

**Figure 1:**
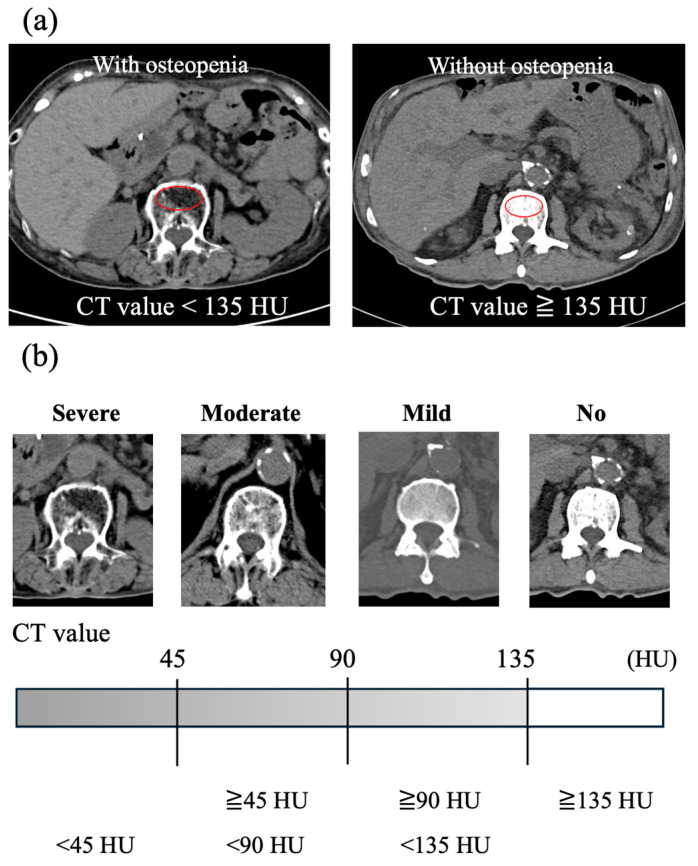
Trabecular L1 CT attenuation value for bone mineral density assessment using CT scan images. (**a**) Examples of axial CT images at the L1 vertebral level in patients with and without osteopenia. The red circle indicates the region of interest for measuring the average CT attenuation of the vertebral trabecular bone. (**b**) Severity of low bone mineral density according to the average CT value of the vertebral trabecular bone. CT, computed tomography; HU, Hounsfield units.

We assessed the presence or absence of osteopenia on the CT scan image taken before TAVI. BMD was evaluated by measuring the trabecular attenuation of the first lumbar vertebra (L1). In accordance with previous reports, low BMD was defined as a CT value of lower than 135 Hounsfield units (HU), i.e., osteopenia [7]. Trabecular CT attenuation was measured using the ShadeQuest/ViewR-DG ver. 1.29 medical imaging workstation (Fujifilm, Tokyo, Japan). Examples are shown in Figure 1a. Since there are no definitive reports on how to classify the severity of osteopenia, we divided the range below 135 HU into three equal segments to define mild (90 HU≥, <135 HU), moderate (45 HU≥, <90 HU), and severe (<45 HU) osteopenia (Figure 1b).

Vertebral fractures were identified using the whole-body CT images used for BMD measurement; deformities of Genant classification Grade 2 or higher were defined as vertebral fractures. To assess sarcopenia, the psoas muscle area at the L4 level was measured on the same CT images, and sarcopenia was defined as values of 20.3 cm^2^ or less in men and 11.8 cm^2^ or less in women [8].

### 2.3. Assessment of Clinical Outcomes

Early clinical outcomes, including technical success, device success, and early safety, were assessed using the Valve Academic Research Consortium 3 (VARC-3) criteria [9]. For long-term outcomes, cardiac events were defined as cardiac death, readmission due to heart failure, aortic valve reintervention, newly required pacemaker implantation, cardiogenic stroke, and other cardiac complications related to TAVI. Cardiac death was defined as death due to cardiac disease or sudden death. All study participants were followed until the end of the study period. The mean follow-up period was 2.4 ± 2.1 years (2.3 ± 2.0 years in patients with osteopenia and 3.2 ± 2.7 years in those without osteopenia).

### 2.4. Statistical Analysis

Continuous data are expressed as mean ± standard deviation and were evaluated using Student’s *t*-test. Categorical data were evaluated using the χ2 or Fisher’s exact test, as appropriate. When the sample size was large, with expected frequencies of 5 or more, the χ2 test was used; otherwise, Fisher’s exact test was applied.

Time-to-event analyses, including overall survival, freedom from cardiac events, and freedom from cardiac death, were performed using the Kaplan–Meier method with the log-rank test. Independent risk factors for overall survival after TAVI were analyzed using a Cox regression hazard model. Multivariable analyses were performed using stepwise regression with forward selection. Variables with *p* < 0.10 from univariable analyses were included in the multivariable models.

Using the Japanese abridged life tables published by the Ministry of Health, Labour and Welfare in Japan [10], predicted survival was derived from sex- and age-matched Japanese population data. A statistically significant difference between the estimated and predicted survival curves was determined by calculating the *z*-value from the cumulative survival rates and their standard errors, as estimated by the Greenwood method [11]. Finally, the *z*-value was used to compute the *p*-value for the difference between the two survival curves. Statistical analyses were performed using JMP ver. 16 (SAS Institute, Cary, NC, USA). Statistical significance was set at *p* < 0.05.

## 3. Results

### 3.1. Baseline Characteristics and Procedural Parameters

Table 1 shows the patients’ background characteristics. Patients with osteopenia had a higher proportion of females and those with lower height, weight, and body surface area than those without osteopenia. Additionally, patients with osteopenia had significantly lower hemoglobin levels and were more likely to have a history of vertebral fractures. A box plot with dot overlay of hemoglobin values is shown in Supplementary Figure S1.

The procedural parameters are shown in Table 2. The approach sites and types of transcatheter heart valves used did not differ significantly between the two groups. Details of the transcatheter heart valves used are shown in Table S1. No significant differences in other surgical factors were observed between the two groups.

### 3.2. Early Clinical Outcomes

The early clinical outcomes are detailed in Table 3. Among the 411 cases, there was only one in-hospital death (in the osteopenia group). Serious adverse events—such as in-hospital death, conversion to open-heart surgery, annulus rupture, and aortic dissection—occurred only in patients with osteopenia but at a very low frequency, and there was no significant difference in incidence between those with and without osteopenia. The hospital stay tended to be longer in patients with osteopenia than in those without osteopenia (11.1 days vs. 8.2 days, *p* = 0.06), and the intensive care unit (ICU) stay was significantly longer in patients with osteopenia (1.8 days vs. 1.4 days, *p* = 0.04).

Regarding the composite endpoints defined by VARC-3, technical success was 97.9% in the osteopenia group and 98.5% in the non-osteopenia group, with no significant difference between the groups. Similarly, device success was 84.7% in the osteopenia group and 84.0% in the non-osteopenia group, and early safety was 64.0% and 75.3%, respectively. Neither showed a significant difference between the groups.

### 3.3. Long-Term Clinical Outcomes

**Figure 2:**
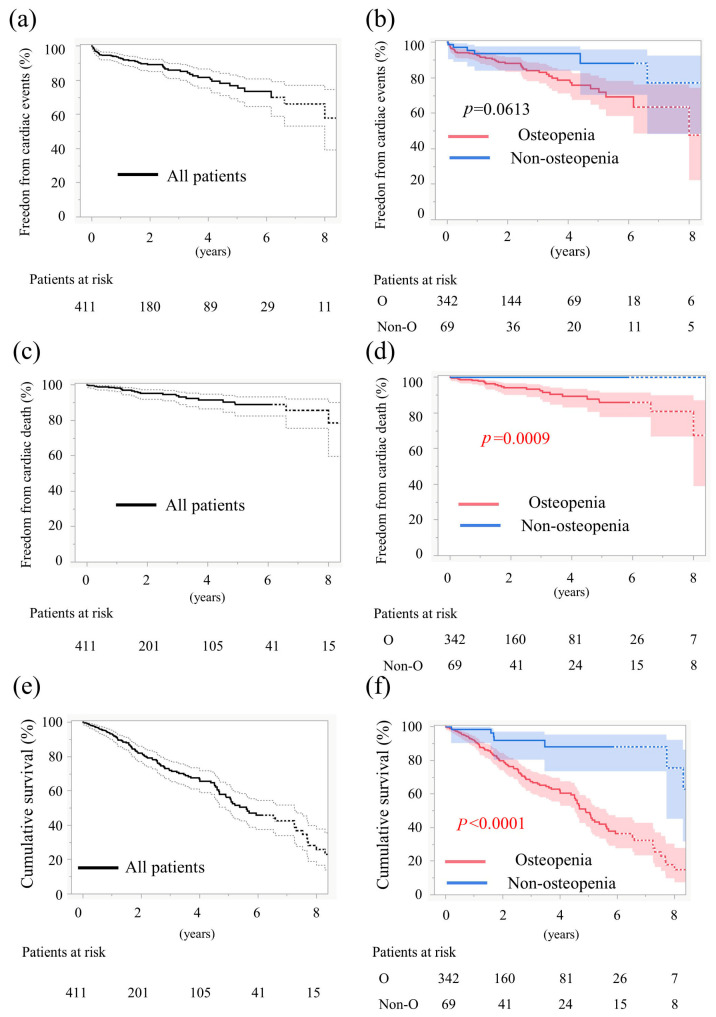
Time-to-event data 1. (**a**) Curves for freedom from cardiac events in all patients. (**b**) Curves for freedom from cardiac events stratified by osteopenia. (**c**) Curves for freedom from cardiac death rate in all patients. (**d**) Curves for freedom from cardiac death rate stratified by osteopenia. (**e**) Curves for cumulative survival in all patients. (**f**) Curves for cumulative survival stratified by osteopenia. Solid lines show the estimated probabilities; the shaded bands in (**b**,**d**,**f**) and the dashed envelopes in (**a**,**c**,**e**) indicate 95% simultaneous CIs (Greenwood, log–log). Numbers at risk are shown below each panel; *p* values are from log-rank tests.

The 6-year cardiac event-free rate was 69.0% in the osteopenia group and 87.9% in the non-osteopenia group, indicating a tendency toward poorer outcomes in patients with osteopenia (*p* = 0.06, log-rank, Figure 2a,b). In the osteopenia group, the breakdown of cardiovascular events was as follows: 30 cases of rehospitalization due to heart failure, 8 cases of sudden cardiac death, 6 cases of new permanent pacemaker implantation, 1 case of acute myocardial infarction, 1 case of aortic valve reintervention, 1 case of valve thrombosis, 1 case of prosthetic valve endocarditis, and 2 cases due to other causes. In the non-osteopenia group, cardiovascular events included 2 cases of rehospitalization due to heart failure, 2 cases of new permanent pacemaker implantation, and 1 case of acute myocardial infarction. The 6-year cardiac-death-free survival rate was 85.7% in patients with osteopenia and 100% in those without osteopenia, indicating a significantly worse outcome in the osteopenia group (*p* = 0.0009, log-rank, Figure 2c,d). Overall survival at 6 years was 36.2% in patients with osteopenia and 88.1% in those without osteopenia, indicating a significantly worse outcome in the osteopenia group (*p* < 0.0001, log-rank, Figure 2e,f).

**Figure 3:**
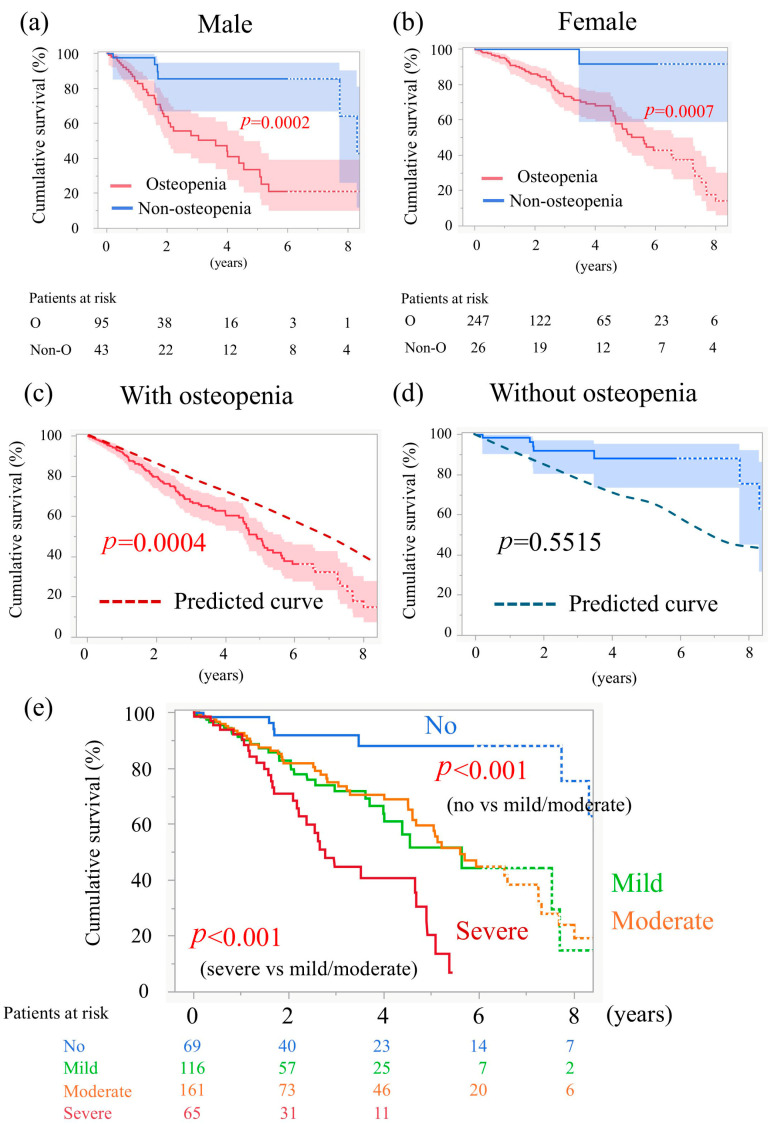
Time-to-event data 2. (**a**) Curves for cumulative survival of males, stratified by osteopenia. (**b**) Curves for cumulative survival of females, stratified by osteopenia. (**c**) Cumulative survival of patients with osteopenia in this study and predicted survival, calculated relative to a sex- and age-matched Japanese general population. (**d**) Cumulative survival of patients without osteopenia in this study and predicted survival. (**e**) Curves for cumulative survival stratified by the severity of osteopenia.

We also examined overall survival stratified by sex. Among male TAVI patients, the 6-year survival rate was 20.8% for those with osteopenia and 85.5% for those without osteopenia (*p* = 0.0002, log-rank, Figure 3a). Among female TAVI patients, the corresponding rates were 42.6% with osteopenia and 91.6% without osteopenia (*p* = 0.0007, log-rank, Figure 3b). In both sexes, patients with osteopenia had a significantly worse prognosis.

### 3.4. Comparison with the General Japanese Population

We compared the 6-year survival of the study patients with the predicted survival rates derived from the Japanese abridged life tables published by the Ministry of Health, Labour and Welfare [10]. In patients with osteopenia, the 6-year survival rate was 36.2%, while the age- and sex-matched survival rate in the Japanese general population was 54.9%. Thus, the prognosis of patients with osteopenia in this study was significantly worse (*p* = 0.0004; Figure 3c). In contrast, the prognosis of patients without osteopenia was comparable to that of the age- and sex-matched general population (*p* = 0.55; Figure 3d).

### 3.5. Mortality Predictors After TAVI

We used a Cox proportional hazards model to analyze predictive factors for mortality after TAVI (Table 4). In the univariate analysis, male sex, cerebrovascular disease, sarcopenia and osteopenia, which showed hazard ratios of 1 or higher, were identified as poor prognostic factors. Higher serum albumin, hemoglobin, and eGFR, which showed hazard ratios of 1 or lower, were favorable prognostic factors. In the multivariate analysis, sarcopenia was no longer a poor prognostic factor, whereas male sex, cerebrovascular disease and osteopenia remained independent predictors of poor prognosis (male: hazard ratio 2.54, 95% confidence interval 1.62–3.97, *p* < 0.0001; cerebrovascular disease: hazard ratio 1.77, 95% confidence interval 1.11–2.82, *p* = 0.016; osteopenia: hazard ratio 6.75, 95% confidence interval 2.96–15.38, *p* < 0.0001).

### 3.6. Severity of Low BMD and Long-Term Survival

Table S2 shows the patient background characteristics according to the severity of low BMD. There were no differences in age among the severity groups. However, the greater the severity, the higher was the proportion of female patients and the lower were the height, weight, and body surface area. The Clinical Frailty Scale was also higher in patients with more severely low BMD. The proportion of vertebral fractures increased with severity, and in the most severe group, approximately 70% of patients had evidence of compression fractures.

The 5-year survival rates were 88.1% for those without osteopenia, 51.5% for mild osteopenia, 59.5% for moderate osteopenia, and 20.2% for severe osteopenia (Figure 3e). The prognosis for mild and moderate osteopenia was equivalent. Compared to patients without osteopenia, both mild and moderate osteopenia were associated with a significantly worse prognosis (*p* < 0.001, Figure 3e). Furthermore, the prognosis of patients with severe osteopenia was worse than that of those with moderate osteopenia (*p* < 0.001, Figure 3e).

## 4. Discussion

This study is one of the few reports to investigate the relationship between BMD and clinical outcomes after TAVI in patients with aortic stenosis. The study findings can be summarized as follows. Patients with osteopenia were predominantly female and tended to have shorter stature, lower body weight, and lower body surface area. Regarding early clinical outcomes, although the incidence of severe complications in patients with osteopenia did not differ from those without, ICU and hospital stays were longer. Long-term clinical outcomes in patients with osteopenia were unfavorable, with higher rates of late cardiac events and cardiac deaths, leading to lower overall survival. Overall survival was particularly poor among patients with severe osteopenia.

Osteopenia is generally considered more prevalent among women. Consistent with this, in our cohort women accounted for 72% of the osteopenia group, whereas the corresponding proportion in the non-osteopenia group was 37.6%. Because sex itself could influence prognosis independently of osteopenia, we examined survival stratified by sex (Figure 3a,b). Nevertheless, in both women and men, patients with osteopenia had poorer outcomes. Furthermore, in the multivariable analysis of risk factors for post-TAVI mortality (Table 3), male sex and osteopenia emerged as independent predictors, whereas female sex was not a risk factor. Taken together, these findings indicate that osteopenia is an independent adverse prognostic factor

In the present study, comorbid osteopenia was observed in approximately 83% of the study population. Age-related decline in BMD is a common phenomenon among older adults, and a substantial proportion of individuals in their 80s, irrespective of sex, are considered to experience some degree of bone loss—either osteopenia or osteoporosis. According to a prior epidemiological study conducted in Japan, osteoporosis was identified in 48.9% of women aged 70 years and older [12]. The mean age of the patients included in the present study was 85.4 years, indicating a predominantly older cohort. Osteoporosis is characterized by a markedly reduced BMD, whereas osteopenia is defined as a decrease in BMD below the normal range. Consequently, the diagnostic threshold for osteopenia is comparatively less stringent than that for osteoporosis. Accordingly, the high prevalence of osteopenia (83%) observed in this cohort is considered appropriate and consistent with the demographic characteristics of the study population.

TAVI was first covered by Japanese health insurance in 2013, and the number of procedures increased significantly thereafter [13]. Additionally, indications have broadened to include low-risk patients, and this trend shows no signs of slowing. However, according to a Japan Transcatheter Valve Therapies (JTVT) report, most TAVI patients in Japan are still older individuals aged approximately 80 years (data available only on the JTVT members-only website). Inevitably, physical changes accompany aging in these older patients, and accurately understanding these changes is vital in geriatric medicine. Decline in muscle strength and bone density are common age-related changes [5]. Bone and muscle regulate each other through mechanical stress and hormones (such as vitamin D, growth hormone, and estrogen), indicating a close relationship between sarcopenia and osteopenia. While there are numerous studies examining sarcopenia in relation to TAVI outcomes [14], research on osteopenia is limited. There have been reports in Japan indicating that TAVI patients with a high risk of osteoporosis tend to have poorer outcomes. That study investigated the relationship between the Osteoporosis Self-Assessment Tool—calculated using a simple formula [(body weight in kilograms − age in years) × 0.2]—and prognosis in TAVI patients [15,16]. However, it focused solely on the risk of low BMD rather than directly measuring bone density, as in the present study. To the best of our knowledge, this is the first report to directly measure bone density in Japanese TAVI patients and investigate its impact on outcomes.

TAVI patients undergo whole-body CT scans preoperatively; therefore, using CT-based diagnosis of osteopenia in these patients is reasonable. Recent studies have shown that decreased bone density (such as that in osteoporosis) not only increases the risk of fractures but is also closely associated with cardiovascular disease [4,5,17]. Low BMD has been reported to exacerbate vascular calcification and increase cardiac workload [16]. In postmenopausal women, reduced estrogen levels simultaneously lead to decreased BMD and progression of arteriosclerosis. Because estrogen exerts vasodilatory and anti-inflammatory effects, its decline is a risk factor for cardiovascular events. In this study, patients with osteopenia tended to experience more cardiac events and had a higher rate of cardiac death in the long term (Figure 2b,d). Additionally, patients with osteopenia had poor prognoses regardless of sex (Figure 3a,b). Furthermore, in the multivariate analysis, sarcopenia—which has previously been reported as a risk factor for mortality after TAVI—was not identified as a risk factor, whereas osteopenia emerged as the sole risk factor. These findings suggest that osteopenia may be a powerful predictor of poor prognosis in Japanese TAVI patients.

In patients with decreased BMD, a primary concern is how to manage treatment after TAVI. Drugs that inhibit osteoclast activity—such as bisphosphonates, receptor activator of nuclear factor κB ligand (RANKL) inhibitors, and zoledronate—carry a serious risk of osteonecrosis of the jaw, making them difficult to introduce without caution. Although calcium or vitamin D supplementation can be started relatively easily, there is currently no evidence that these supplements reduce cardiovascular events on their own [18]. While observational studies suggest that low vitamin D levels are associated with increased cardiovascular risk, evidence from randomized trials indicating that vitamin D supplementation reduces this risk remains insufficient [19]. Furthermore, some reports indicate that calcium supplementation alone increases the risk of myocardial infarction and stroke by approximately 15% [20]. In contrast, current evidence indicates that dietary calcium intake within the range of 200–1500 mg/day does not significantly increase cardiovascular risk [21]. Exercise therapy, meanwhile, has been reported to reduce the risk of falls and improve BMD [22]. Early initiation of rehabilitation is recommended after surgical or transcatheter valve replacement to promote physical recovery before discharge and facilitate sustained functional improvement thereafter. Some reports indicate that early postoperative exercise intervention leads to rapid and sustained improvements in work capacity [23]. However, patients with frailty or multiple comorbidities face greater challenges in continuing rehabilitation, necessitating a customized program that begins with low-intensity exercises and gradually increases the load in stages. Consequently, ensuring adherence to dietary and exercise therapy is likely to form the cornerstone of treatment after TAVI.

Another aspect of this study’s findings deserves attention. Patients without osteopenia demonstrated clearly favorable outcomes, particularly among women. Although the follow-up data were derived from only 26 patients, their survival rate at 9 years was 91.6% (Figure 3b). Consequently, a single TAVI may not be sufficient for such patients to fully address aortic stenosis. Hence, the initial TAVI procedure should be performed with the potential need for a subsequent aortic stenosis intervention in mind. For older female patients without osteopenia and with good physical function, actively choosing SAVR is also a therapeutic option.

During the study period (2014–2024), the indications for TAVI broadened and both device technology and procedural techniques advanced markedly. To evaluate whether generational differences in TAVI therapy and procedural advances influenced prognosis, we compared outcomes by treatment era. The results are presented in Table S3. In this analysis, patients were significantly older in the later era, but other baseline characteristics (sex, STS score, Clinical Frailty Scale, and co-existent osteopenia) were similar between groups. Procedurally, the early era was characterized by more frequent use of transapical access and balloon-expandable valves; these differences reflect the evolution toward lower-profile devices and the timing of regulatory approvals in Japan. Early-safety achievement tended to be lower in the early era, likely owing to the higher use of transapical access. Despite these differences, the comparable prevalence of baseline osteopenia suggests that era-related procedural changes had little impact on the overall study conclusions.

This study has some limitations. First, it was a single-center retrospective study with a small sample size. CT-based diagnosis served as an alternative to dual-energy X-ray absorptiometry, which is the gold standard for measuring bone density. Second, prognosis was evaluated based on the severity of BMD reduction. As there is no universally established definition for the severity of bone loss, we adopted a tertile-based classification. However, this methodological choice represents one of the study’s limitations. Third, as this is an observational study, it is impossible to definitively prove a causal relationship—i.e., whether decreased bone density directly worsens prognosis or whether an overall health condition leading to decreased bone density (e.g., frailty or comorbidities) ultimately contributes to poor outcomes. Because of the retrospective observational design, we cannot establish that low bone density is a causal factor for post-TAVI mortality. Low bone density may instead be a marker of frailty or comorbidity, with those underlying factors determining prognosis. Residual confounding from unmeasured variables—such as nutritional status, physical function, disorders of bone metabolism, and pharmacotherapies—may remain despite adjustment. Fourth, we did not investigate how indicating or withholding osteoporosis treatments (such as bisphosphonates, activated vitamin D, or RANKL inhibitors) might influence prognosis in patients with low BMD. Finally, the study population had a mean age of 85 years. Care for older adults is strongly shaped by ethical views, attitudes toward end-of-life, and religious/cultural norms, which vary substantially across countries; thus, applying these findings outside Japan requires particular caution.

## 5. Conclusions

The study findings demonstrated that long-term outcomes were poor among patients with low BMD. These findings may assist in the stratification of patients undergoing TAVI. To validate the study results, confirmation in large-scale, multicenter, prospective randomized controlled trials is warranted.

## Figures and Tables

**Table 1:** Baseline characteristics.

Variables	Overall (*n* = 411)	With Osteopenia (*n* = 342)	Without Osteopenia (*n* = 69)	*p*-Value
Age, years	85.4 ± 4.9	85.9 ± 4.7	84.5 ± 5.7	0.0897
Female sex *n* (%)	273 (66.4)	247 (72.2)	26 (37.6)	**<0.0001**
Height, cm	149 ± 9	148 ± 9	154 ± 9	**<0.0001**
Weight, kg	50.0 ± 9.5	49.4 ± 9.6	53.1 ± 8.5	**0.0031**
BSA, kg/m^2^	1.42 ± 0.16	1.41 ± 0.15	1.49 ± 0.15	**<0.0001**
Medical history				
Hypertension, *n* (%)	350 (85.1)	289 (84.5)	61 (88.4)	0.3929
Dyslipidemia, *n* (%)	234 (56.9)	196 (57.3)	38 (55.0)	0.7324
Diabetes mellitus, *n* (%)	93 (22.6)	73 (21.3)	20 (28.9)	0.1764
Cerebral vascular disease, *n* (%)	81 (19.7)	67 (19.5)	14 (20.2)	0.5718
Ischemic heart disease, *n* (%)	130 (31.6)	99 (28.9)	31 (44.9)	**0.0109**
Previous cardiac surgery	26 (6.3)	17 (4.9)	9 (13.0)	**0.0221**
Permanent pacemaker, *n* (%)	31 (7.5)	22 (6.4)	9 (13.0)	0.0779
Atrial fibrillation, *n* (%)	51 (12.4)	43 (12.5)	8 (11.5)	0.8206
COPD, *n* (%)	38 (9.2)	29 (8.4)	9 (13.0)	0.2524
Hemodialysis, *n* (%)	6 (1.4)	3 (0.9)	3 (4.3)	**0.0413**
Malignancy, *n* (%)	11 (2.6)	10 (2.9)	1 (1.4)	0.6992
Sarcopenia, *n* (%)	284 (69.0)	233 (68.1)	51 (73.9)	0.3367
Calculated risk scores				
JapanSCORE, %	8.3 ± 7.5	8.2 ± 7.2	9.1 ± 9.0	0.3750
STS-PROM, %	8.3 ± 5.9	8.4 ± 6.1	7.9 ± 4.8	0.5571
EuroSCORE II, %	5.4 ± 5.2	5.4 ± 5.0	5.5 ± 6.1	0.8324
Clinical frailty scale	4.0 ± 1.0	4.0 ± 1.0	3.8 ± 0.9	0.0836
NYHA class	2.3 ± 0.6	2.3 ± 0.6	2.2 ± 0.5	0.7041
Echo parameters (Pre-TAVI)				
Aortic valve area, cm^2^	0.64 ± 0.16	0.64 ± 0.16	0.66 ± 0.17	0.3477
Aortic valve mean PG, mmHg	50.0 ± 17.2	50.0 ± 17.7	48.2 ± 14.3	0.3358
Peak systolic velocity, m/s	4.6 ± 0.7	4.6 ± 0.7	4.5 ± 0.5	0.3724
LVEF, %	61.5 ± 12.6	62.0 ± 12.3	59.3 ± 13.7	0.1104
AR ≥ moderate, *n* (%)	50 (12.1)	43 (12.5)	7 (10.1)	0.5682
Laboratory data				
eGFR, mL/min/1.73 m^2^	49.1 ± 17.2	49.5 ± 17.1	46.8 ± 17.4	0.2572
Hemoglobin, g/dL	11.0 ± 1.5	10.9 ± 1.5	11.4 ± 1.5	**0.0116**
Albumin, g/dL	3.6 ± 0.4	3.6 ± 0.4	3.6 ± 0.4	0.7943
BNP, pg/mL	418 ± 631	417 ± 624	421 ± 670	0.9666
Factors related to bone health				
BMD of L1 trabecular bone (HU)	89.7 ± 45.6	74.7 ± 31.7	164.1 ± 27.2	**<0.0001**
History of vertebral fracture, *n* (%)	144 (35.0)	137 (40.0)	7 (10.1)	**<0.0001**
Steroid use, *n* (%)	36 (8.7)	28 (8.1)	8 (11.5)	0.3776
Bisphosphonate agent use, *n* (%)	29 (7.0)	26 (7.6)	3 (4.3)	0.4448
Vitamin D drug use, *n* (%)	62 (15.0)	54 (15.7)	8 (11.5)	0.3609

*p*-values presented in **bold** indicate statistical significance. BSA, body surface area; BMD, bone mineral density; HU, Hounsfield units; NYHA, New York Heart Association; COPD, chronic obstructive pulmonary disease; STS-PROM, Society of Thoracic Surgeons predictive risk of mortality; LVEF, left ventricular ejection fraction; AR, Aortic regurgitation; BNP, brain natriuretic peptide.

**Table 2:** TAVI procedural parameters.

Variables	Overall (*n* = 411)	With Osteopenia (*n* = 342)	Without Osteopenia (*n* = 171)	*p*-Value
Approach site, *n* (%)				
Femoral	328 (79.8)	270 (78.9)	58 (84.0)	
Non-femoral	83 (20.2)	72 (21.1)	11 (16.0)	0.3347
Details of non-femoral, *n*				
Apical	31	27	4	
Aorta	26	24	2	
Subclavian	14	11	3	
Iliac	12	10	2	
Type of THV, *n* (%)				
Balloon expandable	219 (53.3)	177 (51.8)	42 (60.9)	
Self-expandable	192 (46.7)	165 (48.2)	27 (39.1)	0.1662
Operation time, min	130 ± 44	131 ± 46	124 ± 36	0.2367
Contrast medium, min	98 ± 50	98 ± 48	101 ± 60	0.6290
Fluoroscopy time, min	42 ± 15	42 ± 15	42 ± 15	0.6921
Blood loss, g	120 ± 156	124 ± 164	100 ± 104	0.2348
Blood transfusion, *n* (%)	140 (34.0)	121 (35.3)	19 (27.5)	0.2089
PCPS use, *n* (%)	16 (3.8)	12 (3.5)	4 (5.7)	0.3229

TAVI, transcatheter aortic valve implantation; THV, transcatheter heart valve; PCPS, percutaneous cardiopulmonary support.

**Table 3:** Early clinical outcomes.

Variables	Overall (*n* = 411)	With Osteopenia (*n* = 342)	Without Osteopenia (*n* = 69)	*p*-Value
30-day outcome, *n* (%)				
In-hospital death	1 (0.2)	1 (0.3)	0	>0.999
All-cause mortality	2 (0.4)	2 (0.5)	0	>0.999
Cardiovascular mortality	1 (0.2)	1 (0.3)	0	>0.999
Conversion to open surgery	2 (0.4)	2 (0.5)	0	>0.999
Annulus rupture	1 (0.2)	1(0.3)	0	>0.999
Aortic dissection	4 (0.9)	4 (1.1)	0	>0.999
Access-related minor vascular complication	2 (0.4)	1 (0.3)	1 (1.4)	0.3079
Peri-procedural MI	1 (0.2)	1 (0.2)	0	>0.999
Second THV	3 (0.7)	2 (0.5)	1 (1.4)	0.4247
Unplanned use of PCPS	4 (0.9)	3 (0.8)	1 (1.4)	0.5220
Life-threatening bleeding	8 (1.9)	8 (2.3)	0	0.2425
Cardiac tamponade requiring drainage	2 (0.4)	1 (0.3)	1 (1.4)	0.3079
Stroke	9 (2.1)	9 (2.1)	0	0.3702
Disabling stroke	5 (1.2)	5 (1.4)	0	0.5950
Acute kidney injury—stage 2 or 3	1 (0.2)	1 (0.3)	0	>0.999
New pacemaker implantation	33 (8.0)	30 (8.7)	3 (4.3)	0.3296
Prosthesis-patient mismatch (≥severe)	15 (3.6)	12 (3.5)	3 (4.3)	0.7250
Paravalvular leakage (≥moderate)	36 (8.7)	31 (9.0)	5 (7.2)	0.6261
Hospital stay, days	10.0 ± 12.0	11.1 ± 12.9	8.2 ± 5.0	0.0652
ICU stay, days	1.7 ± 2.1	1.8 ± 2.2	1.4 ± 0.9	**0.0451**
Composite endpoint defined by VARC-3 criteria, *n* (%)				
Technical success	403 (98.0)	335 (97.9)	68 (98.5)	>0.999
Device success	348 (84.6)	290 (84.7)	58 (84.0)	0.8768
Early safety	271 (65.6)	219 (64.0)	52 (75.3)	0.0701

*p*-values presented in **bold** indicate statistical significance. MI, myocardial infarction; PCPS, percutaneous cardiopulmonary support; THV, transcatheter heart valve.

**Table 4:** Predictors of mortality after TAVI.

	Univariate Analysis			Multivariate Analysis		
Variables	Hazard Ratio	(95% Confidence Interval)	*p*-Value	Hazard Ratio	(95% Confidence Interval)	*p*-Value
Age	1.018	(0.983–1.056)	0.312			
Male sex	1.542	(1.029–2.238)	**0.022**	2.540	(1.623–3.976)	**<0.0001**
STS-PROM	1.009	(0.973–1.042)	0.570			
EuroSCORE II	0.970	(0.921–1.008)	0.189			
JapanSCORE	0.990	(0.960–1.016)	0.542			
NYHA class	1.176	(0.884–1.541)	0.250			
Clinical frailty scale	1.144	(0.973–1.338)	0.101			
LVEF	1.012	(0.960–1.028)	0.107			
BNP	1.000	(0.999–1.000)	0.820			
Serum albumin	0.461	(0.298–0.717)	**0.0006**	0.701	(0.424–1.167)	0.170
Hemoglobin	0.816	(0.722–0.920)	**0.001**	0.888	(0.770–1.022)	0.101
eGFR	0.982	(0.969–0.995)	**0.007**	0.985	(0.972–0.998)	**0.0** **30**
Hypertension	0.630	(0.369–1.076)	0.091			
Dyslipidemia	0.639	(0.444–0.919)	0.015	0.749	(0.509–1.102)	0.142
Diabetes Mellitus	0.8326	(0.554–1.311)	0.468			
Hemodialysis	6.875	(0.897–52.667)	0.063	6.481	(0.741–56.657)	0.091
ASO	1.569	(0.999–2.464)	0.051	1.276	(0.772–2104)	0.340
COPD	1.125	(0.546–2.316)	0.749			
CVD	1.770	(1.106–2.481)	**0.014**	1.770	(1.110–2.825)	**0.016**
Sarcopenia	1.536	(1.024–2.302)	**0.037**	1.223	(0.780–1.918)	0.380
Osteopenia	5.253	(2.424–11.385)	**<0.0001**	6.752	(2.964–15.380)	**<0.0001**

*p*-values presented in **bold** indicate statistical significance. ASO, arteriosclerosis obliterans; BNP, brain natriuretic peptide; COPD, chronic obstructive pulmonary disease; CVD, cerebrovascular disease; LVEF, left ventricular ejection fraction; STS-PROM, Society of Thoracic Surgeons predictive risk of mortality.

## Data Availability

The data generated from this study may be shared upon reasonable request with the corresponding author.

## Supplementary Materials

The following supporting information can be downloaded at: https://www.mdpi.com/article/10.3390/jcm14207182/s1, Table S1: Details of the transcatheter heart valves used; Table S2: Baseline characteristics according to the severity of low BMD; Table S3: Comparison of baseline characteristics, procedural parameters and 30-day outcomes in patients undergoing TAVI during the first and second half of the study period. Figure S1: Box plot with dot overlay for Hemoglobin.

## Abbreviations

The following abbreviations are used in this manuscript:

BMD	Bone mineral density
CI	Confidence interval
HU	Hounsfield units
HR	Hazard ratio
ICU	Intensive care unit
JTVT	Japan Transcatheter Valve Therapies
RANKL	Receptor activator of nuclear factor κB ligand
SAVR	Surgical aortic valve replacement
TAVI	Transcatheter aortic valve implantation
VARC-3	Valve Academic Research Consortium 3
