# Supplementary material for: Impact of Computed Tomography-Defined Osteopenia on Outcomes of Transcatheter Aortic Valve Implantation: A Single-Center Retrospective Study
Source: J Clin Med. 2025 Oct 11;14(20):7182. doi: 10.3390/jcm14207182 (PMC12564642; doi:10.3390/jcm14207182)

Table S1. Details of the transcatheter heart valves used

| **Type of THV (Valve size)** | **Overall (n = 411)** | **With osteopenia (n = 342)** | **Without osteopenia (n = 171)** |
|---|---|---|---|
| Balloon expandable, n | | | |
| SapienXT (20/23/26/29 mm) | 58 (2/41/14/0) | 51 (2/36/12/0) | 7 (0/5/2/0) |
| Sapien3 (20/23/26/29 mm) | 132 (12/83/31/6) | 103 (8/68/24/3) | 29 (4/15/7/3) |
| Sapien3 Ultra RESILIA | 29 (7/12/10/0) | 23 (7/10/6/0) | 6 (0/2/4/0) |
| Self-expandable, n | | | |
| CoreValve (23/26/29/34 mm) | 15 (0/10/5/0) | 13 (0/10/3/0) | 2 (0/0/2/0) |
| EvolutR (23/26/29/34 mm) | 21 (3/11/6/1) | 18 (2/10/6/0) | 3 (1/1/0/1) |
| EvolutPRO (23/26/29/34 mm) | 25 (0/15/9/1) | 18 (0/11/6/1) | 7 (0/4/3/0) |
| EvolutPRO+ (23/26/29/34 mm) | 71 (6/41/21/3) | 64 (6/36/19/3) | 7 (0/5/2/0) |
| EvolutFX (23/26/29/34 mm) | 57 (6/29/17/5) | 51 (5/27/15/4) | 6 (1/2/2/1) |
| Navitor (23/25/27/29 mm) | 3 (0/0/1/2) | 1 (0/0/0/1) | 2 (0/0/1/1) |

Table S2. Baseline characteristics according to the severity of low BMD

| Variables | No (n = 69) | Mild (n = 116) | Moderate (n = 161) | Severe (n=65) | p-value |
|---|---|---|---|---|---|
| Age, years | 84.5 ± 5.7 | 85.6 ± 5.1 | 85.2 ± 4.7 | 86.4±3.9 | 0.1302 |
| Female sex n, (%) | 26 (37.6) | 71 (61.2) | 122 (75.7) | 54 (83.0) | **＜0.0001** |
| Height, cm | 154 ± 9 | 150 ± 8 | 148 ± 9 | 145±8 | **<0.0001** |
| Weight, kg | 53.1 ± 8.5 | 51.7 ± 9.5 | 48.7 ± 9.4 | 47.1±9.4 | **<0.0001** |
| BSA, kg/m$^2$ | 1.49 ± 0.15 | 1.45 ± 0.15 | 1.40 ± 0.15 | 1.36±0.15 | **<0.0001** |
| Sarcopenia, n (%) | 51 (73.9) | 83 (71.5) | 106 (65.8) | 44 (67.6) | 0.5825 |
| Clinical frailty scale | 3.8 ± 0.9 | 3.8 ± 0.8 | 4.1 ± 1.0 | 4.2±1.0 | **0.0408** |
| Factors related to bone health | | | | | |
| BMD of L1 trabecular bone (HU) | 164.1 ± 12.7 | 109.6 ± 12.1 | 68.5 ± 12.7 | 28.0±13.7 | **<0.0001** |
| History of vertebral fracture, n (%) | 7 (10.1) | 32 (27.5) | 60 (37.2) | 45 (69.2) | **<0.0001** |
| Steroid use, n (%) | 8 (11.5) | 14 (12.0) | 10 (6.2) | 4 (6.1) | 0.2481 |
| Bisphosphonate agent use, n (%) | 3 (4.3) | 10 (8.6) | 12 (7.4) | 4 (6.1) | 0.7021 |
| Vitamin D use, n (%) | 8 (11.5) | 18 (15.2) | 29 (18.0) | 7 (10.7) | 0.4271 |

BMD, bone mineral density; BSA, body surface area.

P-values presented in **bold** indicate statistical significance.

Table S3. Comparison of baseline characteristics, procedural parameters and 30-day outcomes in patients undergoing TAVI during the first and second half of the study period.

| Variables | First 5 years 2014-2019 (n = 138) | Second 5 years 2019-2024 (n = 273) | p-value |
|---|---|---|---|
| Baseline characteristics | - | | |
| Age, years | 84.7±5.4 | 85.7±4.6 | **0.0400** |
| Female sex n (%) | 92 (66.6) | 181 (66.3) | >0.999 |
| STS-PROM, % | 7.9±4.6 | 8.5±6.5 | 0.319 |
| Clinical frailty scale | 4.1±1.1 | 3.9±0.9 | 0.081 |
| Osteopenia, n (%) | 115 (83.3) | 226 (82.7) | >0.999 |
| TAVI prodedure paratemeter | | | |
| Trans-femoral approach | 103 (74.6) | 225 (82.4) | 0.063 |
| Trans apical approach | 27 (19.5) | 4 (1.4) | **<0.0001** |
| Type of THV, n (%) | | | **<0.0001** |
| Balloon expandable | 123 (89.1) | 96 (35.2) | |
| Self expandable | 15 (10.9) | 177 (64.8) | |
| 30-day outcome, n (%) | | | |
| In-hospital death | 0 | 1 (0.3) | >0.999 |
| All-cause mortality | 1 (0.7) | 1 (0.3) | >0.999 |
| Cardiovascular mortality | 0 | 1 (0.3) | >0.999 |
| Conversion to open surgery | 2 (1.4) | 0 | 0.112 |
| Annulus rupture | 1(0.7) | 0 | 0.335 |
| Aortic dissection | 2 (1.4) | 2 (0.7) | 0.605 |
| Access-related vascular complication | 0 | 2 (0.7) | 0.552 |
| Peri-procedural MI | 1 (0.7) | 0 | 0.335 |
| Second THV | 2 (1.4) | 1 (0.3) | 0.262 |
| Unplanned use of PCPS | 1 (0.7) | 3 (1.0) | >0.999 |
| Life-threatening bleeding | 3 (2.1) | 5 (1.8) | >0.999 |
| Cardiac tamponade requiring drainage | 1 (0.7) | 1 (0.3) | >0.999 |
| Stroke | 5 (3.6) | 4 (1.4) | 0.170 |
| Disabling stroke | 2 (1.4) | 3 (1.0) | >0.999 |
| Acute kidney injury - stage 2 or 3 | 0 | 1 (0.3) | >0.999 |
| New pacemaker implantation | 11 (7.9) | 22 (8.0) | >0.999 |
| Prosthesis-patient mismatch - ≥severe | 4 (2.8) | 11 (4.0) | 0.781 |
| Paravalvular leakage - ≥moderate | 36 (7.9) | 25 (9.1) | 0.853 |
| Hospital stay, days | 13.3 ± 15.7 | 9.2 ± 9.3 | **0.001** |
| ICU stay, days | 1.9 ± 1.1 | 1.6 ± 2.4 | 0.275 |
| Composite endpoint defined by VARC-3 criteria, n (%) | | | |
| Technical success | 134 (97.1) | 269 (98.5) | 0.435 |
| Device success | 116 (84.0) | 232 (84.9) | 0.884 |
| Early safety | 81 (58.6) | 190 (69.5) | **0.036** |

P-values presented in bold indicate statistical significance. PCPS, percutaneous cardiopulmonary support; STS-PROM, Society of Thoracic Surgeons predictive risk of mortality; THV, transcatheter heart valve.

Figure S1. Box plot with dot overlay for Hemoglobin

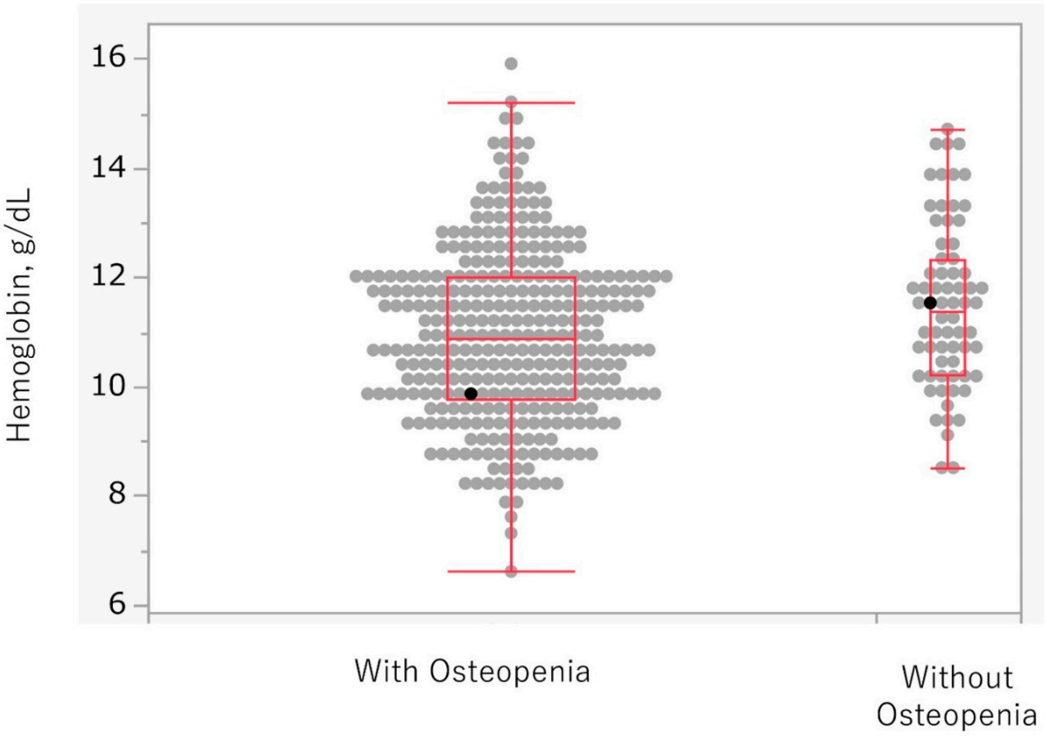

Supplement: Supplementary file 1 [file jcm-14-07182-s001.zip › jcm-3900162-supplementary.pdf]
